# Increasing Nonsedentary Behaviors in University Students Using Text Messages: Randomized Controlled Trial

**DOI:** 10.2196/mhealth.5411

**Published:** 2016-08-19

**Authors:** Emma Cotten, Harry Prapavessis

**Affiliations:** ^1^ Exercise and Health Psychology Laboratory School of Kinesiology Western University London, ON Canada

**Keywords:** sedentary behaviour, prolonged sitting, text messages, self-efficacy, university students, breaks from sitting, light intensity physical activity, moderate intensity physical activity

## Abstract

**Background:**

Sedentary behavior (SB) has been linked to many health problems such as type 2 diabetes and heart disease. Increasing the length and frequency of breaks from sitting and increasing the time spent standing and engaged in light and moderate physical activity are ways to decrease SB. Text message-based interventions have succeeded in aiding smoking cessation and increase both physical activity and healthy eating, but they have not been shown to reduce SB.

**Objective:**

The primary purpose of this pilot study was to determine the effectiveness of a text message-based intervention in increasing nonsedentary behaviors in university students. A secondary purpose was to (1) determine whether the intervention could enhance self-efficacy beliefs for decreasing SB and (2) whether these efficacious beliefs could predict actual SB.

**Methods:**

Eighty-two university students were recruited via mass emails and randomized into intervention (SB-related text messages) or control (text messages unrelated to SB) groups. Participants received daily text messages scheduled by the researcher encouraging breaks from sitting, standing, light- and moderate-intensity physical activity (PA). They then reported various SBs via Web-based questionnaires at four time points (baseline, 2, 4, and 6 weeks). Self-efficacious beliefs toward taking breaks from sitting and decreasing the amount of time spent sitting were assessed at the same time points.

**Results:**

Last observation carried forward (LOCF) method was used for incomplete data as an intent-to-treat (ITT) analysis (intervention group n=15, control group n=11). Small-to-moderate effects favoring the text intervention group were found at 6 weeks for break frequency -14.64 minutes, break length +.59 minutes, standing +24.30 min/day, light-intensity +74.34 min/day, and moderate-intensity + 9.97 min/day PA. Only light-intensity PA approached significance (*P*=.07). Self-efficacy beliefs also favored the text intervention group and reached significance (*P*=.032) for sitting less. Significant (*P*<.05) relations were found between the self-efficacy constructs and breaks, standing, and light or moderate PA.

**Conclusions:**

Text messages have the potential to increase nonsedentary behaviors in university students. These messages can increase self-efficacy beliefs to take more breaks and reduce sitting time. Efficacious beliefs can predict actual SB and to a lesser extent light- and moderate-intensity PA.

**Trial Registration:**

ClinicalTrials.gov NCT02562937; https://clinicaltrials.gov/ct2/show/NCT02562937 (Archived by WebCite at http://www.webcitation.org/6jVLwXE5M)

## Introduction

Sedentary behaviors, such as screen viewing, reading, and riding in an automobile, can be defined as any waking activity at an energy expenditure of ≤1.5 METs (metabolic equivalents) while in a seated or reclined posture [1]. Many adults are physically inactive, meaning they are not meeting the current recommendations of 150 minutes of moderate to vigorous physical activity (PA) per week [2,3]. However, even those who are meeting these recommendations may still be spending too much time sitting, leading to an increase in health risks associated with sedentary behavior [4]. Researchers have found that prolonged sitting (typically in bouts of 20 minutes or more) can cause higher levels of fasting insulin and can increase an individual’s chance of getting type 2 diabetes, increased waist circumference, lower levels of high-density lipoprotein (HDL) cholesterol, increased levels of C-reactive protein, higher levels of triglycerides, raised 2-hour plasma glucose level, and increased risk of all-cause mortality [5-7]. Apart from cardiometabolic risk factors and an increased risk of all-cause mortality, there is evidence that sedentary behavior is related to cancer risks. A meta-analysis found an increased risk in colon, endometrial, and lung cancer associated with extended sedentary time [8]. Healy and colleagues have examined whether breaks from sitting are associated with reductions to known health risks. In one study, they found that those who took the most breaks from sitting had a smaller waist circumference, lower body mass index, lower levels of triglycerides, and lower 2-hour plasma glucose levels, compared with those who took the least amount of breaks from sitting [9,10]. A later study by Healy and colleagues found an association between breaks from sitting and waist circumference, C-reactive protein, and fasting plasma glucose level, irrespective of total sitting time [6]. Beneficial breaks from sitting in these studies were typically 2-4 minutes in length, for every 20 minutes of sitting, which could lead to future guidelines recommending these types of breaks.

Researchers have looked into what constitutes an effective break from sitting, and have found that although standing is better than sitting, light-intensity PA is the most beneficial [6,9]. Although there are no official recommendations of how long adults should sit, early evidence suggests that sitting for 4 hours or less per day may prevent many of the aforementioned health risks. One study, for instance, found that reducing sitting to less than 3 hours per day could result in a 2-year gain in life expectancy [11]. Women who sat for less than 4 hours per day had a much lower prevalence of depressive symptoms [12] and adults who sat for less than 4 hours, regardless of gender, had a reduction in all-cause mortality [13].

The vast majority of sedentary behavior interventions have been aimed at office workers, and overweight or obese adults; however very few, if any, target university students specifically [14]. Students are an inherent sedentary population as they spend a great deal of their time in either class or studying. Studies have shown that weight gain often occurs during young adulthood [14,15], and those who led a sedentary lifestyle in college remained sedentary 5 or 10 years later [16]. Interventions aiming at this population are therefore worth implementing when attempting to prevent high levels of sedentary behavior and reduce overweight or obesity rates in adults.

Although there have been successful interventions developed to reduce sedentary behavior, very few utilize screen-based technology. Many studies have utilized mobile phones to create interventions for other health behaviors via text messages [17-19]. Text messages allow researchers to conveniently reach a large population, either locally or globally, relatively inexpensively and without consuming a great deal of time by either the researchers or the participants. Some of the health behaviors targeted by this method include improving diet, smoking cessation, diabetes management, and increasing PA levels. A recent study used text messages to improve overall health by targeting diet, PA, smoking, and other behaviors related to blood pressure and body mass index and found significant changes in all measures [20]. These results show promise for text messages being used for lifestyle-change interventions.

We are unaware of any studies that have examined the use of text messages as an intervention to reduce sedentary behaviors or increase nonsedentary behaviors in the student or general population. With respect to the student population a large study found that 96% of American undergraduate students owned a mobile phone [21], which indicates that any text message-based intervention that is aimed at this population should be accessible by the vast majority.

Self-efficacy as a determinant of PA has been extensively studied, and results show that those with higher self-efficacy for PA will spend more time being physically active [22,23]. These findings have been replicated using university students, particularly female students [16,24]. The role self-efficacy plays in reducing sedentary behaviors or increasing nonsedentary behaviors is unknown.

The primary purpose of this pilot study was to determine whether a text message intervention would increase frequency and length of breaks from sitting, time spent standing, and time spent in light- and moderate-intensity PA in university students. A secondary purpose was to determine whether the intervention would increase self-efficacious beliefs regarding frequency and length of break from sitting and total sitting time. Another secondary purpose was to determine if self-efficacious beliefs toward length and frequency of breaks and toward sitting less would be related to actual break behavior, time spent standing, and time spent in light- and moderate-intensity PA. Pilot studies are crucial in areas of new research for obtaining preliminary findings with the use of fewer resources. Pilot studies also provide valuable insight into recruitment, randomization, treatment, and follow-up assessments so that these processes can be repeated successfully with a larger main study [25].

## Methods

### Recruitment

After being approved by the Research Ethics Board of Western University, the study was advertised through emails sent out to various faculties at Western University and students who were interested in the study emailed the researcher to sign up. The study was also advertised through an article in the university newspaper due to the interest of a reporter. Eligibility requirements were as follows: participants had to be between the ages of 18 and 65, be able to read and write in English, own a mobile phone with free unlimited incoming text messages, and be a student of Western University.

### Statistical Analysis

#### Power

No previous research exists to inform a sample size power calculation for sitting behavior following a text-based intervention.

#### Data Exclusion

Due to several extreme outliers, a winsorization technique was used to replace any data points over the 95^th^ percentile with the value of the 95^th^ percentile. A total of 196 data points out of more than 6000 data points in the sedentary and light intensity physical activity (SLIPA) questionnaire were imputed this way (60 in the control group and 136 in the intervention group). This method has been shown as a valid way to treat outliers by several authors [26,27].

#### Primary Outcome Measures

##### Frequency of Breaks

The frequency of breaks taken from sitting was measured by the following question “I currently take a break to get up and move around every ——— minutes I spend sitting.” The options the participants could choose from were as follows: every 30 minutes or less, 45 minutes, 60 minutes, 75 minutes, 90 minutes, 120 minutes, 180 minutes or 240 minutes or more.

##### Length of Breaks

Length of breaks taken from sitting was measured by the following question: “Currently, which number best represents the length of your breaks you usually take from sitting?” The answers included 30 seconds or less, 1 minute, 2 minutes, 3 minutes, 4 minutes, 5 minutes, 10 minutes, or 15 minutes.

##### Standing and Light-Intensity Physical Activity

Time spent standing and time spent doing light-intensity physical activity (LIPA) were measured using items 2, 4, 9, 10, 12, 19 and items 3, 7, 8, 11, 13, 14, respectively, of the SLIPA questionnaire. The SLIPA measures time spent doing typical daily sedentary or light-intensity physical activities. The SLIPA has been validated against ActiGraph GTX3 accelerometers, and the cut off points for sedentary behavior and light-intensity physical activity were anything under 100 counts per minute and 100-1951 counts per minute, respectively [28]. The SLIPA is typically used as a 7-day log; however, to ease participant burden, this study asked participants to fill out the items based on a typical weekday and a typical weekend day. Internal consistency Cronbach alphas for the scale constructs were acceptable (at 6 weeks: standing α=.75; light intensity PA α=.81). Although the SLIPA provides a measure of sedentary behavior, the goal of this text intervention was to directly target and positively change standing and light-intensity physical activity. After careful examination of the sedentary behavior items (items 1, 5, 6, 15, 16, 17, and 18), it became evident that some items were not relevant to the text intervention (eg, driving a car) or overlapped each other (eg, sitting-studying, writing, desk work, typing vs. sitting-using a computer) causing many overestimated data points. For these reasons, this sitting measure was not calculated and used in subsequent analyses.

##### Moderate-Intensity Physical Activity

The short form of the Seven-Day Physical Activity Recall Questionnaire was used to measure current levels of moderate-intensity physical activity (MIPA) [22]. Participants were asked to estimate the number of minutes they spent doing MIPA during the last 7 days. Hard and very hard intensity were also measured; however, only moderate intensity was being targeted by some of the texts in the intervention (ie, “Your challenge for tomorrow is to do 30 squats for every episode of TV you watch”), whereas hard and very hard were not specifically targeted, and thus not analyzed.

#### Secondary Outcome Measures

##### Self-Efficacy

To measure self-efficacy, a purpose-built questionnaire was designed. This questionnaire was comprised of 3 questions, each with several statements. The first being “I am ———% confident I can decrease the amount of time I sit every day by 20 minutes,” with possible answers ranging from 0-100 in intervals of 5%. The question was repeated with 30, 45, 60, 75, and 90 minutes. The second question was “I am ———% confident I can take a break from sitting every 240 minutes” which was repeated for 180, 120, 90, 75, 60, 45 and 30 minutes or less. The third question was “I am ———% confident I can increase the length of my breaks from sitting by 30 seconds,” and was also repeated for 1, 2, 3, 4, 5, 10, and 15 minutes. All questions had the same possible answers. The self-efficacy scales demonstrated acceptable internal consistency.

#### Other Measures

##### Demographics

The following demographic information was obtained: name, age, phone number, sex, level of education (undergraduate, graduate, or other), number of hours in class per week, number of hours at work per week, as well as height and weight in order to calculate body mass index.

#### Intervention

##### Sedentary Behavior-Related Text Messages

The intervention group received text messages twice daily, one in the morning or early afternoon and one in the evening, depending on when they reported not being in class or meetings during the first questionnaire. They received one fact about sedentary behavior at the beginning of each week such as, “By breaking up your sitting time you will reduce your risk of developing Type II diabetes,” and included different health risks outlined by Thorpe and colleagues [28]. They then received various challenges, tips, and reminders throughout the week. The challenges started out easy and directly related to the self-efficacy questions such as, “Your challenge for the next 7 days is to get up every hour for 5 minutes,” and got increasingly harder until they were being challenged to get up every 30 minutes for a 5-minute break. The tips and reminders were sent in between challenges and facts and included ways to decrease sitting, such as, “Get up and set a timer on your phone for 5 minutes and don’t sit down again until the timer ends,” “Get off the bus a stop or two early and walk the rest of the way,” or “Don’t forget to get up every hour today and walk around for 5 minutes.” See Multimedia Appendix 1 for list of text messages.

##### Text Messages Unrelated to Sedentary Behavior

The control group received daily text messages in the evenings about random health or nutrition facts, such as, “Raw pumpkin seeds contain essential fatty acids and beneficial proteins” or “Between 25% to 33% of the population sneeze when they are exposed to light.”

#### Procedure

University students who were interested in the study emailed the researcher to sign up in January and then received a link via email that directed them to the first questionnaire, which was administered through a third party website called SoSCI. Upon completion of the baseline measurements, participants were randomized by the researcher, using computer-generated randomized stratification, into either the intervention group or the control group and were unaware of their group allocation. They were then entered into a contact list on the text-messaging website called “Oh Don’t Forget.” “Oh Don’t Forget,” is a Web-based application that works through “Recess Mobile” to send messages from a computer to mobile phone numbers that are programmed into the application.

All participants began receiving text messages within 3 days of completing the questionnaire. Every participant received the same daily texts as each other participant in their group, with times varying slightly depending on their schedule. After 2 weeks of receiving texts, participants received the link to the second questionnaire in an email and were also reminded via text to complete it. This was repeated at 4 and 6 weeks, respectively. All questionnaires contained the same measures as described previously (except for demographics that were only asked at baseline, and physical activity recall, which was only asked at baseline and at 6 weeks in order to reduce the length of the questionnaires). Upon completion of the final questionnaire the participants were notified that they would no longer be receiving text messages and that the study was completed. Data collection was all done at Western University, beginning in January 2015 and was completed in March 2015. See Multimedia Appendix 2 for the CONSORT EHEALTH checklist [29].

#### Statistical Analyses

##### Primary and Secondary Outcome Analyses

A series of 2 (intervention vs controls) x 4 (time – baseline, 2 weeks, 4 weeks, and 6 weeks) repeated measures analysis of variance (ANOVA) were used to determine if there were any significant time or time by group interaction effects. Bivariate correlations were conducted on the self-efficacy questionnaires and their matching behaviors. Linear regression was used to determine how much of the variance in the behavior could be predicted by the matching self-efficacy questionnaire.

## Results

### Missing Data

**Figure 1:**
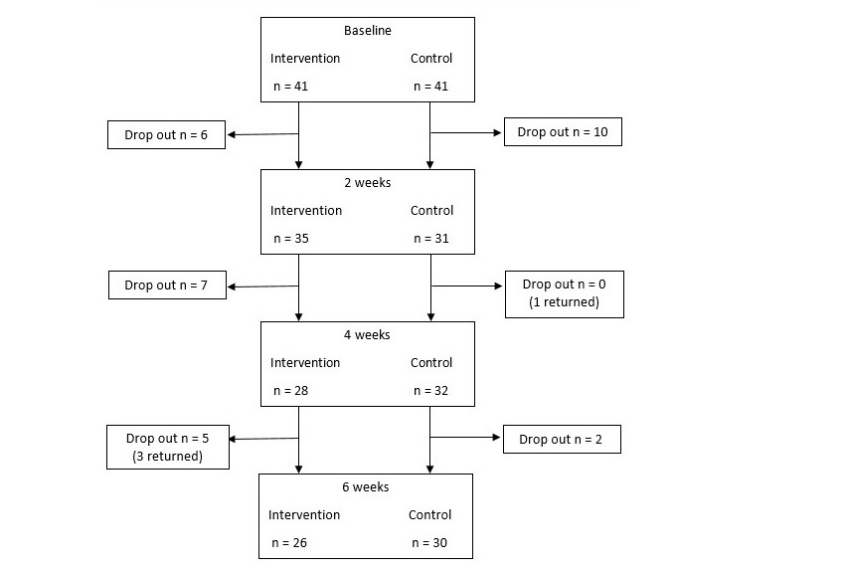
Flow of participants through study.

Last observation carried forward (LOCF) method was used for missing data from dropouts as an intent-to-treat (ITT) analysis. Independent t-tests revealed no significant differences (all P values > .05) between those who gave complete data and those who dropped out at any time points. There were also no significant differences in the demographic variables for those that provided complete versus missing primary outcome data (all P values > .05). In addition, there was no differential loss between treatment groups for those who provided complete end point data (all P values > .05). Taken together, all missing data were considered random. Figure 1 shows dropouts for each group.

**Table 1:** Demographic variables of both groups at baseline.

Demographic variables		Intervention group (n=41)			Control group (n=41)		
		M	SD	%	M	SD	%
Sex (male)				24.4			26.8
Age (years)		21.37	3.60		21.02	4.76	
Body mass index		24.57	3.56		23.22	3.54	
Hours of class per week		15.63	7.42		15.67	6.73	
Hours of work per week		6.33	8.99		5.94	10.35	
**Type of student**							
	Undergraduate			87.8			78
	Graduate student			12.2			17
	Other			0			4.8
**Physical activity**							
	Moderate^a^	184.15	269.40	203.54	183.98
	Hard^a^	94.12	120.63		83.24	121.42	
	Very Hard^a^	93.23	113.23		97.61	131.70	
Days with hard		2.80	2.34		2.93	2.40	
Days with moderate		4.25	3.61		4.34	2.66	

^a^Expressed in minutes per week

### User Statistics

Descriptive statistics for the demographic and physical activity variables are shown in Table 1. Groups were equivalent at baseline for all measures (all P values > .05).

### Evaluation Outcomes

#### Primary Outcomes – Break Frequency and Length, Standing, Light-Intensity Physical Activity and Moderate-Intensity Physical Activity

Descriptive data for the primary outcomes are presented in Multimedia Appendix 3. These data show that the intervention group increased standing by 18.25 min/day, LIPA by 50.07 min/day, MIPA by 13.03 min/day (total increase in PA/standing of 81.35 minutes). The control group decreased standing by 6.05 min/day, decreased light by 24.27 min/day and increased moderate by 3.06 min/day (total net decrease of 27.26 minutes).

There were significant time effects for break frequency: F (3, 78)=6.32, *P*<.001, Wilks’ Λ=0.80, η_ρ_^2^=.20; time spent in light-intensity PA: F (3, 78)=2.75 *P*=0.048, Wilks’ Λ=0.90, η_ρ_^2^=.10; and time spent in moderate-intensity PA: F (3, 80)=5.25, *P*=.025, Wilks’ Λ=0.94, η_ρ_^2^=.06. There were no significant time effects for break length: F (3, 78)=0.73 *P*=.537, Wilks’ Λ=0.97, η_ρ_^2^=.03 or time spent standing: F (3, 78)=0.45, *P*=.715, Wilks’ Λ=.98, η_ρ_^2^=.02.

There were no significant treatment group by time interaction effects for break frequency: F (3, 78)=1.28, *P*=.287, Wilks’ Λ=0.95, η_ρ_^2^=.05; break length: F (3, 78)=0.73 *P*=.629, Wilks’ Λ=0.98, η_ρ_^2^=.02; time spent standing: F (3, 78)=0.72, *P*=.544, Wilks’ Λ=.97, η_ρ_^2^=.03; or time spent in moderate: F (3, 80)=2.01, *P*=.160, Wilks’ Λ=0.98, η_ρ_^2^=.03. However, there was a trend effect for time spent in light: F (3, 78)=2.43 *P*=.071, Wilks’ Λ=0.91, η_ρ_^2^=.09.

#### Secondary Outcomes - Self-Efficacy

Descriptive data for the secondary outcomes are presented in Multimedia Appendix 3. Confidence to increase frequency of breaks increased by 7.74% for the intervention group and by 4.34% for controls. Confidence to increase length of break increased in the intervention group by 0.90% and decreased for the controls by 1.37%. Confidence to decrease sitting time increased by 11.44% for the intervention group and by 6.31% for the controls.

There were significant time effects for confidence to increase break frequency: F (3, 78)=9.79 *P*<.001, Wilks’ Λ=0.73, η_ρ_^2^=.27, confidence to increase break length: F (3, 78)=6.41 *P*=.001, Wilks’ Λ=0.80, η_ρ_^2^=.20 and confidence to sit less: F (3, 78)=8.54 *P*<.000, Wilks’ Λ=0.75, η_ρ_^2^=.25.

There were trend interaction effects for confidence to increase break frequency F (3, 78)=2.52 *P*=.064, Wilks’ Λ=0.91, η_ρ_^2^=.09 and for confidence to increase break length: F (3, 78)=2.06 *P*=.112, Wilks’ Λ=0.93, η_ρ_^2^=.07. There was a significant interaction effect for confidence to sit less: F (3, 78)=3.09 *P*=.032, Wilks’ Λ=0.89, η_ρ_^2^=.11.

#### Associations Between Self-Efficacy and Target Behaviors

Bivariate data for relations between the self-efficacy constructs and the targeted primary outcome variables is presented in Table 2. At 6 weeks, confidence to increase break frequency was significantly related to actual break frequency, actual break length, standing time, LIPA, and MIPA. Confidence to increase break length was significantly related to actual break length, actual break frequency, standing time, LIPA, and MIPA. Confidence to sit less was significantly related to break frequency, break length, standing time, LIPA, and MIPA.

**Table 2:** Correlation between self-efficacy and target behaviors at baseline and 6 weeks^h^

	SE-BFrequency^c^	SE-BLength^d^	SE-SL^e^	Break Frequency	Break Length	Stand	LIPA^f^	MIPA^g^
SE-BFrequency	-	.478^b^	.585^b^	-.408^b^	.310^b^	.171	.157	.172
SE-BLength	.374^b^	-	.637^b^	-.367^b^	.560^b^	.163	.260^a^	.251^a^
SE-SL	.347^b^	.487^b^	-	-.398^b^	.347^b^	.219^a^	.318^b^	.336^b^
Break Frequency	-.576^b^	-.091	-.093	-	-.241^a^	-.128	-.180	-.146
Break Length	.130	.329^b^	.147^b^	-.114	-	-.089	.194	.323^b^
Stand	.198	.130	.125	.079	-.073	-	.693^b^	.305^b^
LIPA	.174	.258	.137	.161	-.100	.665^b^	-	.396^b^
MIPA	.204	.032	.123	.064	-.208	.251^a^	.195	-

^a^*P*<0.05

^b^*P*<0.01

^c^SE-BFrequency: self-efficacy for break frequency

^d^SE-BLength: self-efficacy for break length

^e^SE-SL: self-efficacy for sitting less

^f^LIPA: Light-intensity physical activity

^g^MIPA: Moderate-intensity physical activity

^h^Values below the diagonal are from baseline, numbers above the diagonal are from 6 weeks.

## Discussion

### Summary of Results

The present pilot study aimed to use text messages to increase (1) the frequency and length of breaks from sitting, (2) the amount of time spent standing, and (3) and the amount of time engaged in light- and moderate-intensity physical activity. The study also aimed to increase self-efficacy for breaks and reduce overall sitting time. Overall small to moderate effects that did not reach significance were found that consistently favored the text intervention group for all primary outcome behaviors. Irrespective of behavior, the largest difference between treatment groups occurred at 6 weeks. Moderate to large effects that reached significance were also found consistently favoring the text intervention group for all self-efficacy constructs measured. Again, irrespective of self-efficacy measure, the largest difference between treatment conditions occurred at 6 weeks. Finally, significant relations were found when correspondence was high between the self-efficacious constructs and the primary outcome behaviors. Relations between measures were stronger at week 6 than at baseline. Beyond these general observations, the following specific issues warrant commentary.

### Principal Results

#### Frequency and Length of Breaks

Frequency of break resulted in a net difference of 14.64 minutes between groups, favoring the intervention group. Although this difference is not statistically significant, it could still be clinically meaningful as the intervention group is getting up to move around more frequently.

Length of break from sitting resulted in a difference of 0.59 minutes between groups. This small nonsignificant increase is not surprising because the intervention was aiming at taking 3-6 minute breaks for every 30 minutes of sitting, or 6-10 minute breaks every hour. The intervention group was above 6 minutes every hour, and thus, behaving consistently with recommendations of previous work [9,10].

#### Standing, Light, and Moderate Physical Activity

Time spent (1) standing resulted in net difference of 24.30 minutes per day, (2) doing LIPA resulted in a net difference of 74.34 minutes per day, and (3) doing MIPA resulted in a net difference of 69.78 minutes per week (9.97 minutes per day). Overall, the net differences were moderate in size and favored the intervention group and approached significance only for LIPA. These results are not surprising as the text messages focused more on replacing sitting with light to moderate physical activity rather than standing. Failure for the net differences highlighted above to reach statistical significance is likely due to the study being underpowered due to the small sample size and the variances of responses being widely dispersed around the means of the targeted nonsedentary behaviors.

Previous studies have shown a range of increased standing time from 57 minutes per day [30] to 127 minutes per day [31]. This study only increased standing by 18.25 minutes per day. Studies that focused on increasing LIPA were successful in increasing it by 31 minutes per day after 4 weeks [32], 21 minutes per day after 6 months [33], and 39 minutes per day after 1 year [34]. This study was able to increase LIPA by 50.07 minutes per day. One study that looked at standing and LIPA increased standing by 57 minutes per day and LIPA by 38 minutes per day for a total increase of 95 minutes after 9 months [30]. The change seen in the current intervention had a combined increase in standing and PA of 81 minutes per day. Most studies focused on standing or LIPA; however, a study by Carr et al also measured moderate-intensity physical activity and found an increase of 8.8 minutes per day, along with a 2.2 minute increase in vigorous PA and 6.4 minute increase in LIPA per day [35,36]. The current study observed an increase of 13.03 minutes per day of moderate physical activity. Taken together, our findings provide evidence that text messaging as a way to increase standing, LIPA and MIPA is, for the most part, in line with other interventions.

#### Self-Efficacy

Confidence to sit resulted in a net difference of 5.13% that reached statistical significance. Confidence to take more frequent breaks resulted in a net difference of 3.4%. Confidence to increase length of breaks from sitting resulted in a net difference 2.27%. Overall, the net differences were small and favored the intervention group.

At 6 weeks, the self-efficacy measures were significantly related to their matching behaviors. These findings underscore the importance of scale correspondence between the cognition and the targeted behavior. Confidence to sit less had significant relationships with breaks, standing, LIPA, and MIPA at baseline. This suggests that those who are more confident in being able to sit less will take longer and more frequent breaks, and spend more time standing, in LIPA and MIPA. It also could mean that those WHO demonstrate these behaviors are more confident in sitting less. Future work should shed light on whether efficacious beliefs towards breaks and sitting less are antecedents or consequences of sitting less behaviors. Future work might also focus on developing scales that measure efficacious beliefs toward standing as well as using existing scales that measure efficacious beliefs towards LIPA and MIPA [22,23].

### Strengths, Limitations and Future Directions

There are several strengths associated with the present pilot study. These include the randomized control design with equal contact time, the inexpensive and user-friendly text messaging system used, tailoring the text messages to each individual’s schedule, and matching the text messages with the targeted nonsedentary behaviors and efficacious cognitions. Another strength is the study’s scalability. This study was conducted using a sample of university students; however, it could easily be replicated using many other populations. Since mobile phones are so common, anyone who uses one daily could benefit from this type of intervention. It could be adapted to specific groups, such as office workers, by having messages scheduled during their lunch breaks, or in the evenings, to remind them to get up and move around, rather than just sit in front of their computer or television. It could also be used for retired adults, to keep them active once they no longer have the daily routines that they had during the years they spent working. Using messages similar to those from this intervention could be combined with existing technology to create other interventions that utilize fitness trackers or mobile phone apps.

The main limitation for this study was the use of a subjective self-report measure of sedentary behavior. Although the SLIPA questionnaire has been shown to be a valid and reliable measure in the past, it was not problem-free in this study. For instance, many people overestimated how much time they spend doing various activities representing sitting time (which was shown when their days would add up to many more than 24 hours) that were either not relevant to the text intervention or overlapped each other. Hence, a sitting time measure was not calculated and used in subsequent analyses. The use of an objective measurement tool such as an accelerometer with a built in inclinometer would allow for more accurate data as well as data that are more valuable. If such a device was worn throughout the study, it would give the exact amount of time that was displaced from inactivity to other behaviors. It would also allow the researchers to observe if the participants were actually utilizing the prompts from the texts by checking the data at the time the texts were received. If a text was sent that told them to get up and move around for 5 minutes, the researchers could examine the device data at that time and see if the participant did indeed move around for 5 minutes right away, if they were delayed, or if they did not move at all.

A further limitation was that there were a high number of dropouts from both groups over the 6 weeks. Fortunately, as mentioned previously, there was no real differential loss between groups and no significant differences in demographics between those who dropped out and those who remained in the study. Another limitation that was highlighted above was the small sample size that prevented many of the net differences that favored the text message intervention to reach statistical significance. The small sample size, paired with the specific population of university students, makes it hard to determine generalizability, thus more research should be done to look into other populations with larger samples. Finally, the study was advertised as a way to reduce sedentary behavior, and thus, participants in both groups self-selected into the trial because they were highly motivated to change this behavior. This may partially explain why larger net differences were not found between intervention and control group participants.

### Conclusions

The present study provides preliminary evidence that facts, tips, reminders, and challenges delivered in the form of text messages have potential to increase nonsedentary behaviors, and in particular, light-intensity physical activity in university students. These text messages appear to enhance self-efficacious beliefs about taking more breaks and reducing overall sitting time. Self-efficacious beliefs are also associated with nonsedentary behavior (ie, breaks from sitting) and to a lesser extent light- and moderate-intensity physical activity. An RCT that uses a larger sample size, objective measures of sedentary and nonsedentary behavior, and assesses related efficacious cognitions over a longer period of time is warranted.

## Appendix

Multimedia Appendix 1 List of text messages.

Multimedia Appendix 2 CONSORT-EHEALTH checklist V1.6.2 [29].

Multimedia Appendix 3 Tables (Results).

## Abbreviations

ANOVA: analysis of variance
HDL: high-density lipoprotein
ITT analysis: intent-to-treat analysis
JMIR: Journal of Medical Internet Research
LIPA: light-intensity physical activity
LOCF: last observation carried forward
MIPA: moderate-intensity physical activity
PA: physical activity
RCT: randomized controlled trial
SLIPA: sedentary and light-intensity physical activity
SB: sedentary behavior
