# Supplementary material for: Increasing Nonsedentary Behaviors in University Students Using Text Messages: Randomized Controlled Trial
Source: JMIR Mhealth Uhealth. 2016 Aug 19;4(3):e99. doi: 10.2196/mhealth.5411 (PMC5010648; doi:10.2196/mhealth.5411)
Supplement: Multimedia Appendix 1 [file mhealth_v4i3e99_app1.pdf]

Multimedia Appendix 1

List of text messages sent to intervention group

Hi Name12345, welcome to the study. You will be receiving texts with challenges, tips and reminders every day for the next 6 weeks. Each week the challenges will become more difficult in hopes of ending up closer to recommended levels of sitting and frequency of breaks.

Wondering why reducing sitting time is so important? By breaking up your sitting time, you can reduce your risk of heart disease.

For the next seven days, your challenge is to make sure outside of class and meetings you get up at least once every hour. To make it easier, try to stand up and move around every hour on the hour.

If you've been sitting for more than an hour it is time to get up and move around! Right now!

Keep remembering to get up every hour. Try walking around or doing some light stretching while standing.

Hey Name12345, take a break from all that studying... Or TV watching. Get up and stretch your legs.

On top of getting up every hour, your challenge for today is to replace 20 minutes of sitting with walking. Walk to school instead of bussing. Or take a walk through campus after class.

Have you gone for a walk yet today? It's not too late if you haven't!

Just because it's the weekend doesn't mean it's time to be totally lazy. Keep breaking up your sitting every hour and try to replace an hour of usual sitting time with walking this weekend. Split it up into 30 mins each day, or go on one nice long walk. Your choice!

Are you watching TV? Be sure to take a break between episodes to get up and move around!

Make sure to get that walk in today and to avoid sitting for more than an hour at a time. You got this!

Your challenge for tomorrow is to do 30 squats for every episode of TV you watch.

Choose your shows wisely!

Hey Name12345, make sure you are keeping track of any TV you watch today so you get enough squats in.

Tomorrow be sure to replace 20 minutes of sitting with walking again. If it's easier, you can break it up into smaller amounts.

Are those legs sore from all the squats yesterday? Stretching is a great way to fix that!

Get up and stretch or walk around every hour.

Need a tip to sit less? Try walking to school or the store if you live close enough, or if you drive, park further away from where you are going. It'll add some steps to your day and give you some nice fresh air!

Here is another reason to sit less: taking a break from sitting to walk around or do some light stretching can help strengthen your bones.

Your 7 day challenge is to get up for at least 3 minutes every hour. Start a timer, put on a 3 minute song or if you are really bored, count to 240 slowly before sitting down again. By the end of the day you should know what 3 mins feels like without any timer!

Good morning Name12345, make sure you are getting up every hour and staying up for 3 minutes today!

It's almost the weekend. To celebrate, go for a nice, long, 40 minute walk tomorrow.

It'll help you destress from the week!

TGIF, am I right? Keep getting up every hour for 3 mins today, and don't forget about that walk! It'll be good for you.

This weekend aim to replace an hour and a half of sitting time with walking or exercise.

That's only 45 minutes a day! Easy!

Have you been spending a long time on the computer today? For every hour you spend online, try to go for a 20 minute walk. Or skip the computer time completely and just go for a nice long walk today!

Time to get up, especially if you've been sitting for a while! Go run up and down some stairs or do some jumping jacks for a few minutes!

Don't forget about that 45 minutes of exercise today. Maybe some fresh air will help clear your mind for the upcoming week.

Continue breaking up your sitting every hour with at least a 3 minute break for the next few days. Squats, lunges and jumping jacks are all great ways to kill 3 mins! Try a minute of each and see how many you can do.

It's been 2 weeks! Check your e-mail for the next questionnaire, it should only take 20 minutes. Why not do it standing up?

Tomorrow, try to replace as many sedentary activities with active ones! Text or e-mail standing up, take the stairs instead of elevator, stand up on the bus, walk to school, just keep moving!

Reminder to complete the 2 week questionnaire if you have not done so yet!

Hey Name12345, hope you were able to make a lot of active choices today! Keep it up and soon they will become great healthy habits!

You’re going to want to stand up to read this one! Studies show we sit for an average of

7.7 hours/day, with some of us sitting for up to 15 hours in one day! Keep breaking up your sitting time to stay below that average and try to throw in an extra-long walk this weekend.

For the next 7 days aim to get up every 45 minutes outside of class time and stay up for 4 minutes.

Keep up with those 4 min breaks every 45 minutes and on top of that try to replace 60 minutes of sitting a day with exercise. An hour workout would be a great replacement to sitting! Sit less and get fit? Sounds great!

Hey Name12345, are you starting to feel groggy or tired? Try taking a short walk to wake up.

If working out isn't for you and you still need ideas to sit less, try some pick up sports at the gym, doing yoga or even just going for a walk, it'll make you feel so much better to know you are being active!

Get up! Do some jumping jacks, walk around or stretch. Your body will thank you.

For every 45 mins you spend studying (or watching TV) this week, give your brain a break and do some light exercise or walk around.

As Bob Marley says, "Get up, Stand up, Stand up for your health". Okay those aren't quite the lyrics, but you get it. Stand up!

Next time you finish reading a page in your textbook, or next commercial take a break to walk around.

If you ever find yourself sitting for a long time during class or a meeting, consider making up for it by sitting less, later.

Those who sit for 3 hours or more per day watching TV are 64% more likely to die from heart disease. This includes watching TV online!

I hope you didn't think I'd give you a scary fact without a tip on how to avoid it! From now on, your challenge is to increase your breaks from sitting to being 5 minutes long. For every 45 minutes you spend sitting, spend 5 doing something more active.

Here's a tip to decrease your sitting time: if you ride the bus to work or school try standing instead of sitting.

On top of yesterday's challenge, try to replace 80 minutes of time usually spent sitting (before these texts changed your life) with walking or dancing, or exercise of any sort! Bonus points for working up a sweat!

Pick 5 exercises (squats, lunges, jumping jacks, calf raises, push-ups, etc.) and do each one for a minute during one of your breaks. Do this each day for the remainder of the 6 weeks and see how many you can do by the end! Practice makes perfect!

Watching a show? Make it a rule to get up for 5 minutes between episodes, whether it's on TV, online or Netflix!

This weekend try to get in 90 minutes of walking or exercise each day during times that would normally be spent sitting. Go to the gym for an hour, break it up into 20 minute chunks, do whatever you want to make it happen!

If you've been sitting for a while it's time to get up! Move around for 5 minutes before you sit down again.

How's that weekend exercise coming? It may seem daunting but you'll feel much better afterwards!

Hope you had a great, active break. Please check your e-mail for the week 4 questionnaire!

For every half hour of time spent online today, on your phone or computer, do 5 minutes of exercise! Pick whatever form of exercise you want! Try something that'll get your heart rate up!

If you haven't filled out the questionnaire yet please do so today!

Got a break between classes? Go for a walk around campus, or explore some of the underground passages instead of sitting down.

Breaking up your sitting time can reduce your risk for certain types of cancer. You have more control over our health than you think!

With there only being 2 weeks left of these texts it's time to make sure you are on track with the recommendations for sitting. From now on, try to get up every 30 minutes and stay up for 5 minutes. Do some light exercise or cleaning, anything that keeps you moving!

For the next 7 days your challenge is to replace 80 minutes a day of sitting with any type of exercise. If you were already doing this for an hour, that only leaves 20 more mins during the day to replace! That's not bad.

How has taking more frequent breaks from sitting been? Hopefully it makes you feel energized and less lazy!

This weekend try to replace 2 hours a day of sitting with any type of activity. Maybe your room needs some tidying up, it's never too early to do some spring cleaning.

Weekends usually mean a whole lot more sitting, try to limit it as much as possible.

You can still have fun and relax, just find a way to do it without sitting!

Got some errands to run this weekend? One way to replace sitting is to walk to as many places as you can instead of driving or bussing.

If you are still having trouble finding a way to stop sitting, grab a friend or a roommate and go for a walk together, or to the gym!

Tomorrow your challenge is to do 5 squats for every minute you spend on Facebook, or Instagram, etc. Maybe it'll make your more active and get you off those websites quicker!

Don't forget about your squat challenge. Monitor your time closely and if you underestimate you're only lying to yourself!

For the last 7 days your challenge is to replace 100 minutes of sitting with walking or activity each day. This may seem like a lot, but if you have been keeping up it's only 20 more minutes a day than last week! That's nothing!

If you don't have time for a long walk every day, break up the 100 minutes throughout the day. Your body will thank you for getting up and moving more often since that is what it was made to do!

Is that 100 minutes still feeling overwhelming? If you get up every 30 minutes for a 5 min break during your free time, that counts for half of the 100 minutes in 5 hours. For the other 50 minutes go for a walk or go workout!

Hey name12345, it's been 6 weeks, please check your e-mail for the final questionnaire.

Time to keep up these goals on your own. Hopefully they have become habits by now, and if not, just keep practicing them until they are automatic! Your health is worth the effort.

If you haven’t done the questionnaire yet please do it ASAP. Once you’re done, why not go stretch your legs on a nice walk outside?
