# Supplementary material for: Increasing Nonsedentary Behaviors in University Students Using Text Messages: Randomized Controlled Trial
Source: JMIR Mhealth Uhealth. 2016 Aug 19;4(3):e99. doi: 10.2196/mhealth.5411 (PMC5010648; doi:10.2196/mhealth.5411)
Supplement: Multimedia Appendix 3 [file mhealth_v4i3e99_app3.pdf]

Multimedia Appendix 2

Results including Means, Standard Deviations, and 95% Confidence Intervals for Frequency of Breaks, Length of Break, Standing, LIPA and MIPA time, as well as self-efficacy measures at each time point.

Table 5: Means, Standard Deviations, and 95% Confidence Intervals for Frequency of Breaks at Each Time Point. M (minutes)

Time	Intervention Group			Control Group		
	M	SD	95% CI	M	SD	95% CI
Baseline	81.95	45.39	[67.63, 96.28]	77.56	41.47	[64.47, 90.65]
2 weeks	65.49	20.88	[58.90, 72.08]	64.76	21.06	[58.11, 71.41]
4 weeks	60.00	23.72	[52.51, 67.49]	60.20	22.99	[52.94, 67.45]
6 weeks	58.90	20.78	[52.34, 65.46]	69.15	36.57	[57.60, 80.69]

Table 6: Means, Standard Deviations, and 95% Confidence Intervals for Length of Break at Each Time Point. M (minutes)

Time	Intervention Group			Control Group		
	M	SD	95% CI	M	SD	95% CI
Baseline	6.71	4.08	[5.42, 7.99]	6.37	4.71	[4.88, 7.85]
2 weeks	6.76	4.14	[5.45, 8.06]	6.68	4.14	[5.38, 7.99]
4 weeks	7.12	3.64	[5.97, 8.27]	6.93	4.16	[5.61, 8.24]
6 weeks	7.49	4.39	[6.10, 8.86]	6.56	4.18	[5.24, 7.88]

Table 7: Means, Standard Deviations, and 95% Confidence Intervals for Time Spent Standing at Each Time Point. M (minutes/day)

Time	Intervention Group			Control Group		
	M	SD	95% CI	M	SD	95% CI
Baseline	517.02	327.04	[413.80, 620.25]	409.34	325.68	[306.54, 512.13]
2 weeks	533.22	392.93	[409.19, 657.25]	408.24	317.00	[308.19, 508.30]
4 weeks	497.88	386.63	[375.84, 619.91]	409.49	323.94	[307.24, 511.74]
6 weeks	535.27	399.22	[409.26, 661.28	403.29	280.02	[314.92, 491.68]

Table 8: Means, Standard Deviations, and 95% Confidence Intervals for Time Spent in Light Intensity Physical Activity per Day at Each Time Point. M (minutes/day)

Time	Intervention Group			Control Group		
	M	SD	95% CI	M	SD	95% CI
Baseline	315.05	150.57	[267.52, 362.58]	297.98	166.02	[245.57, 350.38]
2 weeks	338.83	239.73	[263.16, 414.50]	289.63	164.35	[237.76, 341.51]
4 weeks	378.80	247.77	[300.60, 457.01]	304.41	201.22	[240.90, 367.93]
6 weeks	365.12	235.56	[290.77, 439.47]	273.71	152.96	[225.43, 321.99]

Table 9: Means, Standard Deviations, and 95% Confidence Intervals for Time Spent in Moderate Intensity Physical Activity at Baseline and After 6 weeks. M (minutes/week)

Time	Intervention Group			Control Group		
	M	SD	95% CI	M	SD	95% CI
Baseline	184.15	269.40	[99.11, 269.18]	203.54	183.98	[145.47, 261.61]
6 weeks	275.37	267.04	[191.08, 359.65]	224.98	207.40	[159.51, 290.44]

Table 10: Means, Standard Deviations, and 95% Confidence Intervals for Self-Efficacy of Frequency of Breaks at Each Time Point. M (%)

Time	Intervention Group			Control Group		
	M	SD	95% CI	M	SD	95% CI
Baseline	81.16	14.12	[76.70, 85.62]	77.84	16.22	[72.72, 82.96]
2 weeks	86.06	14.07	[81.62, 90.50]	76.15	18.71	[70.24, 82.06]
4 weeks	89.07	12.43	[85.15, 92.99]	84.52	14.35	[79.98, 89.05]
6 weeks	88.90	12.19	[85.05, 92.75]	82.18	14.50	[77.60, 86.76]

Table 11: Means, Standard Deviations, and 95% Confidence Intervals for Self-Efficacy of Length of Breaks at Each Time Point M (%)

Time	Intervention Group	Control Group

	M	SD	95% CI	M	SD	95% CI
Baseline	85.63	14.40	[81.08, 90.17]	82.39	16.16	[77.29, 87.50]
2 weeks	81.25	17.68	[75.67, 86.83]	78.68	16.91	[73.34, 84.01]
4 weeks	81.85	18.21	[76.10, 87.60]	80.61	16.69	[75.34, 85.88]
6 weeks	86.53	15.11	[81.76, 91.30]	81.02	17.17	[75.60, 86.44]

Table 12: Means, Standard Deviations, and 95% Confidence Intervals for Self-Efficacy of Decreasing Daily Sitting Time at Each Time Point. M (%)

Time	Intervention Group			Control Group		
	M	SD	95% CI	M	SD	95% CI
Baseline	67.42	21.93	[60.42, 74.26]	64.61	21.26	[57.90, 71.32]
2 weeks	67.07	23.58	[59.63, 74.52]	69.11	17.03	[63.73, 74.48]
4 weeks	73.19	21.19	[66.50, 79.88]	69.57	18.12	[63.85, 75.29]
6 weeks	78.86	20.75	[72.31, 85.41]	70.92	18.12	[65.20, 76.64]
